# Insulin Resistance as a Shared Pathophysiological Driver in Neurological Disorders: a Narrative Review

**DOI:** 10.1007/s13668-026-00743-7

**Published:** 2026-02-28

**Authors:** Aslıhan Atar, İrem Nur Şahin Anılgan

**Affiliations:** 1 Department of Nutrition and Dietetics, Faculty of Health Science, Istanbul Beykent University, Istanbul, Turkey; 2 Department of Nutrition and Dietetics, Institute of Health Sciences, Istanbul Medipol University, Istanbul, Turkey; 3 Department of Nutrition and Dietetics, Institute of Health Sciences, Gazi University, Ankara, Turkey

**Keywords:** Insulin resistance, Brain insulin signaling, Neurodegeneration, Neuroinflammation, Synaptic plasticity, Metabolic interventions, Nutrition

## Abstract

**Purpose of Review:**

Insulin resistance has traditionally been viewed as a peripheral metabolic abnormality; however, accumulating evidence indicates that impaired insulin signaling within the central nervous system plays a critical role in the pathogenesis of multiple neurological disorders. The purpose of this narrative review is to synthesize current evidence supporting insulin resistance as a shared pathophysiological driver across neurodegenerative, neurological, and neuropsychiatric conditions, and to evaluate the potential of nutrition- and metabolism-based interventions as modulatory strategies.

**Recent Findings:**

Recent human and experimental studies demonstrate that central insulin resistance disrupts brain energy metabolism, promotes neuroinflammation, impairs synaptic plasticity, and alters neuronal network stability. These mechanisms contribute to disease onset and progression in Alzheimer’s disease, Parkinson’s disease, epilepsy, and mood disorders. Advances in neuroimaging, cerebrospinal fluid biomarkers, and molecular profiling have strengthened the link between impaired insulin signaling and cognitive, behavioral, and affective dysfunction. In parallel, emerging evidence suggests that dietary patterns, energy restriction, ketogenesis, and lifestyle interventions can partially restore insulin sensitivity, improve metabolic flexibility, and mitigate neurobiological vulnerability.

**Summary:**

Insulin resistance should be regarded not merely as a comorbid metabolic condition, but as an active disease-modifying factor in a broad spectrum of neurological disorders. Targeting insulin signaling pathways through personalized nutritional and metabolic interventions represents a promising, modifiable strategy for prevention and adjunctive management. Future research integrating metabolic phenotyping with neurological outcomes will be essential to translate these insights into clinical practice.

## Introduction

Although the brain constitutes only a small fraction of total body weight, it is a metabolically demanding organ responsible for a significant proportion of overall energy consumption. The regulation of neuronal activity, synaptic transmission, plasticity, and cognitive processes relies on precise control of glucose metabolism, mitochondrial function, and hormonal signaling pathways [1, 2]. While the brain was previously considered insulin-independent, recent evidence indicates that insulin exerts substantial neuromodulatory and neurotrophic effects in the central nervous system [3, 4].

**Fig. 1:**
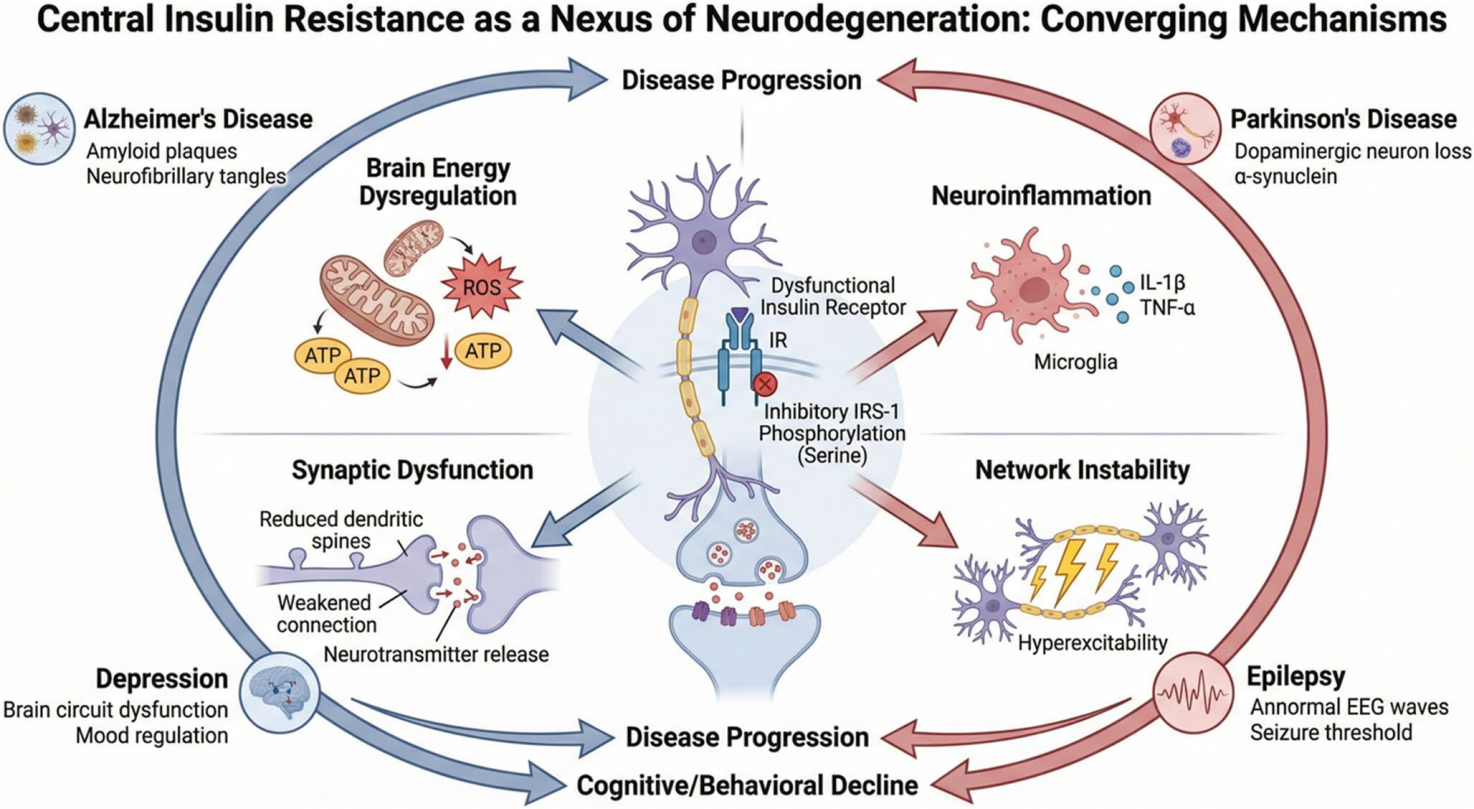
Central insulin resistance as a nexus of neurodegeneration: converging mechanisms

Central insulin resistance has been most extensively studied in Alzheimer’s disease, which is often referred to as “type 3 diabetes” due to the close association between disrupted insulin signaling and hallmark pathological features such as amyloid-β accumulation, tau hyperphosphorylation, and synaptic loss [5, 6]. However, the effects of insulin resistance extend beyond Alzheimer’s disease. Similar impairments in insulin signaling pathways have been identified in Parkinson’s disease, epilepsy, major depressive disorder, and other neurodegenerative or neuropsychiatric conditions [7, 8]. These findings indicate that insulin resistance may serve as a common underlying pathophysiological mechanism across a range of neurological disorders (Fig. 1).

This review examines insulin resistance as a shared pathophysiological mechanism across neurological diseases and synthesizes the existing literature within an integrative framework. It addresses the physiological roles of brain insulin signaling, the cellular and molecular consequences of central insulin resistance, and its associations with various neurological disorders. Furthermore, the review discusses the potential clinical and translational implications of this mechanism for the prevention and treatment of neurological diseases.

## Brain Insulin Signaling and Central Insulin Resistance

Insulin receptors (IRs) are highly expressed in the brain, especially in regions essential for cognitive processing and metabolic integration, including the hippocampus, prefrontal cortex, hypothalamus, and olfactory bulb. These receptors are found in various cell types, predominantly neurons and astrocytes, and are activated by autophosphorylation upon insulin binding [9, 10]. Signal transduction primarily involves two intracellular pathways: the IRS-PI3K-Akt pathway and the MAPK/ERK pathway. The PI3K-Akt pathway is closely linked to the regulation of glucose transporters, synaptic plasticity, neuronal survival, and anti-apoptotic mechanisms [9, 11]. In contrast, the MAPK/ERK pathway is central to gene expression, neuronal differentiation, and long-term synaptic modifications [4, 8]. Through these signaling mechanisms, insulin acts in the brain as both a metabolic regulator and a neuromodulator that supports cognitive processes.

Central insulin resistance refers to diminished responsiveness of brain insulin receptors or downstream signaling components to insulin. This condition can arise independently of peripheral insulin resistance or may develop concurrently. Central insulin resistance is characterized by inhibitory serine phosphorylation of IRS-1, decreased PI3K-Akt pathway activation, and increased oxidative stress and neuroinflammation. These changes impair synaptic plasticity, suppress long-term potentiation, and particularly disrupt hippocampal-dependent memory processes [3, 12]. Collectively, these mechanisms establish central insulin resistance as a common pathophysiological substrate for both neurodegenerative and neuropsychiatric disorders.

Direct assessment of central insulin resistance in humans presents significant methodological challenges. Common approaches include evaluating brain responses with functional magnetic resonance imaging (fMRI) after intranasal insulin administration, measuring cerebral glucose metabolism using positron emission tomography (PET), and analyzing insulin and IRS-1 phosphorylation profiles in cerebrospinal fluid [13, 14]. These methods are constrained by high costs, limited accessibility, and challenges in establishing causality. Additionally, peripheral metabolic status may mask central measurements, and substantial interindividual variability further restricts the generalizability of results [15]. Therefore, there is a pressing need to develop more sensitive and standardized biomarkers for assessing central insulin resistance in human populations.

## Shared Pathophysiological Mechanisms

### Brain Energy Dysregulation

Disruption of brain energy homeostasis is among the earliest and most consistent findings in neurological disorders associated with insulin resistance. Under physiological conditions, insulin indirectly facilitates neuronal glucose uptake, glycolysis, and oxidative phosphorylation, thereby supporting the substantial energy requirements of synaptic activity. In central insulin resistance, diminished insulin receptor-mediated PI3K-Akt signaling reduces neuronal glucose utilization, leading to regional glucose hypometabolism [8]. This hypometabolic pattern, particularly prominent in brain regions integral to cognitive function such as the hippocampus, posterior cingulate cortex, and prefrontal cortex, is detectable prior to the onset of overt clinical symptoms in several neurodegenerative disorders, most notably Alzheimer’s disease [16–18].

Impairments in glucose utilization, as identified by positron emission tomography with fluorodeoxyglucose (FDG-PET), indicate not only reduced neuronal energy supply but also diminished synaptic integrity and network functionality [3]. Energy insufficiency is linked to suppression of long-term potentiation, disruption of neurotransmitter recycling, and decreased cognitive flexibility. In metabolic conditions characterized by insulin resistance, these processes collectively contribute to a shared vulnerability that underlies cognitive decline and neurodegeneration [19].

Mitochondrial stress represents another fundamental mechanism closely associated with glucose hypometabolism. In addition to meeting the high energy demands of neurons, mitochondria are essential for regulating calcium homeostasis, redox balance, and cellular survival signaling pathways [20]. Disrupted insulin signaling impairs mitochondrial biogenesis and dynamics, including the balance between fusion and fission, reduces electron transport chain efficiency, and increases reactive oxygen species production. The resulting oxidative stress further impairs neuronal function through protein oxidation, lipid peroxidation, and mitochondrial DNA damage [21].

Mitochondrial stress is not exclusive to Alzheimer’s disease but is recognized as a common pathological feature across a wide range of neurological and neuropsychiatric disorders, including Parkinson’s disease, epilepsy, and mood disorders. Chronic impairments in energy production diminish neuronal metabolic flexibility, which facilitates the progression of disease-specific pathologies [22]. Therefore, the combined analysis of glucose hypometabolism and mitochondrial stress provides an integrative framework for understanding the widespread, systemic effects of insulin resistance in the brain [23].

### Neuroinflammation

Neuroinflammation constitutes a fundamental pathophysiological process common to neurological disorders associated with insulin resistance. Microglia, the resident immune cells of the central nervous system, perform critical functions under physiological conditions, including synaptic pruning, clearance of cellular debris, and maintenance of neuronal network integrity [24]. In the presence of central insulin resistance, microglia undergo chronic activation and adopt a pro-inflammatory phenotype. Impaired insulin signaling is associated with activation of the NF-κB pathway in microglial cells, increased cytokine production, and disruption of phagocytic homeostasis [25].

Chronic microglial activation induces the release of pro-inflammatory mediators, including interleukin-1β, interleukin-6, and tumor necrosis factor-α, which negatively impact synaptic function and neuronal survival. The resulting inflammatory microenvironment impairs synaptic plasticity in brain regions essential for cognitive networks, such as the hippocampus and cortex, and contributes to accelerated cognitive decline [26]. While amyloid-β accumulation is recognized as a trigger for microglial activation in Alzheimer’s disease, insulin resistance has also been demonstrated to independently modulate and intensify this process, serving as a reinforcing factor [3, 25].

Metabolic inflammation is characterized by the reciprocal interaction between metabolic signals and immune responses, serving as a critical determinant of neuroinflammation in the context of insulin resistance. In systemic metabolic disorders, including obesity, type 2 diabetes, and dyslipidemia, elevated concentrations of free fatty acids, advanced glycation end products, and endotoxins initiate both peripheral and central inflammatory responses [27]. These metabolic signals increase the permeability of the blood–brain barrier, thereby facilitating the entry of peripheral inflammatory mediators into the brain and intensifying microglial activation [28].

Within the brain, metabolic inflammation establishes a vicious feedback loop that further suppresses insulin signaling. Pro-inflammatory cytokines enhance inhibitory phosphorylation of IRS-1, thereby reducing the effectiveness of the PI3K-Akt pathway and deepening central insulin resistance [11]. This self-perpetuating cycle, when combined with energy dysregulation, oxidative stress, and synaptic dysfunction, creates a shared pathological substrate across multiple neurological disorders. Consequently, neuroinflammation should not be regarded merely as a secondary, disease-specific response, but rather as a fundamental mechanism that, together with insulin resistance, drives the onset and progression of neurological disorders [8, 26].

### Synaptic Dysfunction and Excitability

Insulin signaling serves a critical regulatory function in maintaining synaptic plasticity in the central nervous system. Activation of insulin receptors promotes membrane trafficking of α-amino-3-hydroxy-5-methyl-4-isoxazolepropionic acid (AMPA) and N-methyl-D-aspartate (NMDA) receptors at glutamatergic synapses, and facilitates long-term potentiation (LTP) [29]. Central insulin resistance suppresses the PI3K-Akt pathway, leading to decreased synaptic protein synthesis and disrupted receptor phosphorylation homeostasis. These changes directly impair learning and memory, particularly within hippocampal circuits [9, 30].

Synaptic dysfunction is recognized as a major neurobiological predictor of cognitive decline in Alzheimer’s disease [31]. While amyloid-β and tau pathologies exert toxic effects at the synaptic level, insulin resistance further amplifies these effects by creating a permissive environment [32]. Impaired insulin signaling accelerates synaptic loss and weakens compensatory plasticity mechanisms. These findings indicate that synaptic integrity is influenced not only by disease-specific proteinopathies but also by disruptions in metabolic signaling [33].

In addition to its role in synaptic plasticity, insulin acts as a key regulator of neuronal excitability and network balance. Insulin signaling modulates GABAergic and glutamatergic transmission, adjusts action potential thresholds, and regulates ion channel activity [34]. Central insulin resistance disrupts these regulatory processes, resulting in an imbalance between excitation and inhibition within neuronal networks. Consequently, hyperactivity may develop in some circuits, while others experience functional suppression [4, 35].

This imbalance parallels the pathological network hyperactivity observed in epilepsy and Alzheimer’s disease. Increased neuronal firing and synchronization within hippocampal circuits are associated with both cognitive impairment and heightened seizure susceptibility. The promotion of these network-level changes by insulin resistance demonstrates that metabolic dysfunctions can directly affect neuronal excitability. Therefore, synaptic dysfunction and altered neuronal excitability serve as key intermediate mechanisms underlying the widespread impact of insulin resistance across neurological disorders [19, 36].

## Neurological Disorders Associated with Insulin Resistance

### Alzheimer’s Disease

Alzheimer’s disease (AD) is one of the neurological disorders in which central insulin resistance has been most extensively described and clinically characterized. Disruption of brain insulin signaling is strongly associated with declines in episodic memory and executive functions. Postmortem analyses of human brain tissue have demonstrated increased inhibitory serine phosphorylation of insulin receptor substrate-1 (IRS-1) in the hippocampus and cortical regions, a change that negatively correlates with cognitive performance scores [12]. These findings suggest that insulin resistance is not merely a comorbid condition in AD but may serve as a direct determinant of cognitive decline.

Clinical studies indicate that peripheral insulin resistance, type 2 diabetes, and metabolic syndrome increase the risk of developing AD and accelerate its progression. Individuals with insulin resistance display earlier and more extensive cerebral glucose hypometabolism. This metabolic impairment reduces cognitive reserve and limits compensatory mechanisms, thereby facilitating a more rapid onset of clinical symptoms [3, 37].

At the mechanistic level, insulin resistance interacts bidirectionally with the core pathological features of Alzheimer’s disease. Impaired insulin signaling alters amyloid precursor protein (APP) metabolism, resulting in increased production and accumulation of amyloid-β [38]. It also disrupts the balance between kinases and phosphatases that regulate tau protein phosphorylation [39]. Suppression of the PI3K-Akt pathway leads to enhanced activity of glycogen synthase kinase-3β (GSK-3β), which accelerates tau hyperphosphorylation [5, 33]. These molecular changes promote synaptic loss and progressive neuronal degeneration.

Clinically, interventions targeting central insulin signaling, such as intranasal insulin administration, have been shown to transiently improve cognitive performance in specific patient subgroups. Although these findings are heterogeneous, they indicate that insulin resistance may represent a modifiable therapeutic target in Alzheimer’s disease [40]. In this context, Alzheimer’s disease serves as a model disorder for understanding the effects of insulin resistance on cognitive function, providing insights that may be applicable to other neurological conditions.

### Parkinson’s Disease

Parkinson’s disease (PD) is a neurodegenerative disorder marked by progressive loss of nigrostriatal dopaminergic neurons, manifesting as both motor and non-motor symptoms, including cognitive and mood disturbances. Recent evidence indicates that insulin resistance is a significant metabolic stressor that heightens the vulnerability of dopaminergic neurons [41, 42]. Due to their high energy requirements, intense mitochondrial activity, and substantial oxidative stress, dopaminergic neurons are especially prone to metabolic disturbances. Impaired insulin signaling in the brain leads to mitochondrial dysfunction, reduced antioxidant defenses, and decreased cellular stress tolerance in these neurons [4, 7].

Experimental studies demonstrate that insulin receptor signaling regulates dopamine synthesis, release, and reuptake. In central insulin resistance, reductions in striatal dopamine turnover, impaired dopamine transporter (DAT) function, and associated behavioral changes have been observed [43]. These findings indicate that insulin resistance is not solely a comorbid condition but also a pathophysiological factor that directly impairs dopaminergic circuit integrity [4].

Epidemiological data indicate that type 2 diabetes, obesity, and metabolic syndrome are associated with an increased risk of Parkinson’s disease. Individuals with these metabolic disturbances often experience earlier disease onset, faster progression of motor symptoms, and more severe non-motor manifestations. Chronic inflammation and mitochondrial stress linked to insulin resistance may promote α-synuclein accumulation and aggregation, accelerating neurodegeneration [44, 45].

Clinically and translationally, there is increasing interest in therapeutic strategies that target insulin signaling as potential neuroprotective interventions for Parkinson’s disease. Notably, studies of glucagon-like peptide-1 (GLP-1) receptor agonists have demonstrated improvements in both motor and cognitive symptoms. These findings support the perspective that Parkinson’s disease is not only a classical movement disorder but also a metabolically modifiable neurodegenerative process [7, 46].

### Epilepsy

The capacity of neuronal networks to sustain adequate energy supply and maintain ionic homeostasis is a critical determinant of seizure threshold in epilepsy. Metabolic stress, including insulin resistance, inefficient glucose utilization, mitochondrial dysfunction, and increased oxidative burden, can suppress Na⁺/K⁺-ATPase activity, compromise membrane potential stability, and disrupt the balance between inhibitory and excitatory signaling, thereby facilitating neuronal hyperexcitability [47]. In this context, metabolic dysregulation is increasingly recognized not only as a comorbidity but also as a permissive substrate that lowers seizure threshold and promotes epileptogenesis in specific epilepsy phenotypes [48, 49].

Clinical and epidemiological evidence indicates a bidirectional relationship between metabolic risk and epilepsy. Population-based studies have identified associations between type 2 diabetes, glycemic dysregulation, particularly severe hypoglycemic episodes, and the development of epilepsy. Additionally, type 2 diabetes may increase epilepsy risk independently of severe hypoglycemia [50]. Conversely, metabolic syndrome is prevalent among individuals with epilepsy, and certain antiepileptic drugs, such as valproate, can exacerbate metabolic risk profiles. These findings highlight the importance of metabolic risk assessment in the management of epilepsy [51]. Recent large-scale database analyses further support the association between epilepsy and metabolic disorders, including diabetes, obesity, and hypertension [52].

The ketogenic diet (KD) is a well-established metabolic therapy with demonstrated clinical efficacy, particularly in drug-resistant epilepsy. The anticonvulsant effects of KD are attributed to multiple converging mechanisms, including enhanced brain energy efficiency via altered metabolic fuel utilization, improved mitochondrial function, reduced oxidative stress and inflammatory responses, modulation of neuronal membrane excitability, and increased inhibitory tone [48].

In the context of insulin resistance, KD reduces carbohydrate intake and lowers insulin demand, thereby fostering a metabolic environment that may enhance insulin sensitivity in some individuals. This is particularly relevant to the relationship between metabolic stress and seizure threshold in epilepsy, as stable energy metabolism and reduced glycemic variability may mitigate the energy and ionic imbalances that contribute to neuronal hyperexcitability. Furthermore, clinical evidence indicates that dietary interventions reducing glycemic load without inducing full ketosis, such as low–glycemic index treatments, can also decrease seizure frequency. These observations suggest that metabolic and seizure control may share overlapping biological mechanisms [53].

### Depression and Anxiety

While depression and anxiety disorders have historically been attributed to neurotransmitter imbalances and psychosocial factors, recent research has increasingly focused on their associations with metabolic disturbances. Insulin resistance can negatively influence mood regulation by activating the hypothalamic-pituitary-adrenal axis, promoting low-grade chronic inflammation, and suppressing signaling pathways essential for neuroplasticity [54]. Disruption of central insulin signaling may specifically impair monoaminergic systems, such as serotonin and dopamine, thereby heightening susceptibility to depressive and anxiety-related symptoms [8, 55].

Epidemiological evidence demonstrates a bidirectional relationship between type 2 diabetes, insulin resistance, and both major depressive disorder and anxiety disorders. Metabolic inflammation and elevated cytokine levels impair insulin signaling and diminish synaptic plasticity in brain regions responsible for mood regulation [56]. This convergence indicates that, in specific subtypes of depression and anxiety, metabolic phenotypes can influence both disease severity and treatment outcomes [28, 57]. Therefore, insulin resistance should be regarded not only as a comorbid condition in neuropsychiatric disorders but also as a fundamental component of the underlying pathophysiological network.

## Current Evidence in Nutrition and Metabolic Interventions

Dietary patterns play a critical role in modulating insulin sensitivity and central metabolic signaling. The Mediterranean diet, which is rich in monounsaturated fatty acids, dietary fiber, polyphenols, and antioxidants, has been identified as a dietary pattern that enhances both peripheral and central insulin sensitivity [58]. Both observational and interventional studies indicate that greater adherence to the Mediterranean diet correlates with a lower risk of cognitive decline and a more favorable metabolic profile. These benefits are likely mediated by the suppression of inflammation, reduction of oxidative stress, and stabilization of glycemic fluctuations, thereby supporting brain energy homeostasis [59, 60].

Diets characterized by a low glycemic load may help limit the progression of central insulin resistance by stabilizing postprandial insulin responses. Reduced glycemic variability supports a more consistent neuronal energy supply, which may benefit synaptic excitability and cognitive function [61]. This nutritional strategy is considered a rational approach to improving neurological outcomes, especially in individuals with metabolic risk factors [62, 63].

Energy restriction and intermittent fasting regimens are interventions that have been shown to improve insulin signaling and enhance metabolic flexibility. Evidence from animal and human studies demonstrates that energy restriction increases insulin sensitivity, promotes mitochondrial biogenesis, and reduces neuroinflammation [64–66]. These metabolic adaptations may optimize brain energy utilization and help preserve cognitive performance [22].

Ketogenic approaches, while long established in the clinical management of epilepsy, have also attracted growing interest in the context of neurodegenerative and neuropsychiatric disorders. During ketosis, elevated ketone bodies provide an insulin-independent energy source, partially compensating for glucose hypometabolism [67]. In addition, ketogenic diets have been reported to suppress inflammation and oxidative stress and, in some individuals, to improve insulin resistance. Nevertheless, the long-term sustainability of these approaches and their efficacy across different neurological disorders remain heterogeneous and should be carefully evaluated according to patient phenotype [17, 68].

**Fig. 2:**
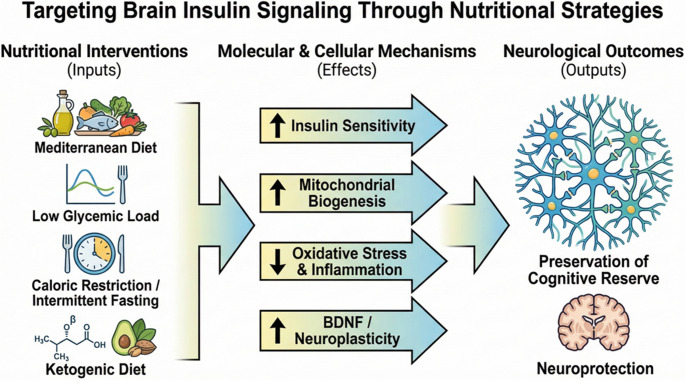
Targeting brain insulin signaling through nutritional strategies

Nutritional interventions may have a limited impact when evaluated independently of other lifestyle factors such as physical activity and sleep patterns. Regular physical activity enhances both peripheral and central insulin sensitivity, promotes the release of neurotrophic factors, and supports synaptic plasticity [69]. In contrast, insufficient sleep and chronic stress may exacerbate insulin resistance, thereby increasing metabolic and neurological vulnerability. Consequently, nutrition-based strategies are likely to exert more meaningful and sustainable effects on insulin resistance-associated neurological disorders when implemented as part of multidimensional lifestyle interventions [8, 22] (Fig. 2).

## Clinical Implications and Future Directions

Current evidence indicates that insulin resistance is not merely a comorbidity in neurological disorders; rather, it acts as an accelerating factor that drives disease onset and progression by inducing energy dysregulation, neuroinflammation, synaptic dysfunction, and network imbalance [70, 71]. This effect may significantly alter the clinical trajectory of neurodegenerative disorders such as Alzheimer’s and Parkinson’s diseases, particularly through early glucose hypometabolism and mitochondrial stress. In epilepsy and certain neuropsychiatric conditions, the central role of metabolic stress in determining seizure threshold and mood regulation further establishes insulin resistance as a shared and clinically significant therapeutic target [47, 72].

Within this context, early identification and longitudinal monitoring of metabolic status provide clear clinical benefits by slowing cognitive decline, reducing symptom severity, and enhancing treatment responsiveness. Insulin resistance constitutes a modifiable risk factor that may account for the heterogeneity observed across neurological disorders.

The effectiveness of nutritional and metabolic interventions in neurological diseases varies substantially according to individual metabolic phenotypes. Factors such as insulin resistance status, glycemic response profiles, body composition, inflammatory burden, and physical activity levels influence responses to dietary patterns, including the Mediterranean diet, low-glycemic load approaches, energy restriction, and ketogenic interventions. This variability underscores the need to move beyond uniform dietary recommendations toward personalized nutritional strategies.

Individuals with elevated metabolic risk are more likely to experience significant neurological benefits from dietary pattern modifications, while effects may be limited in metabolically resilient phenotypes. Therefore, integrating metabolic phenotyping into routine clinical practice is increasingly important as a fundamental aspect of neurological disease management.

A critical priority for future research is the development of reliable, standardized biomarkers to assess central insulin resistance. Comprehensive longitudinal studies that integrate brain imaging, cerebrospinal fluid biomarkers, and peripheral metabolic measurements are essential for clarifying causal relationships. Additionally, randomized controlled trials should evaluate nutritional and lifestyle interventions by measuring metabolic parameters alongside neurological outcomes as primary endpoints.

Recognizing insulin resistance as a shared pathophysiological substrate across neurological disorders highlights the importance of interdisciplinary approaches in future research. These strategies may facilitate the integration of metabolic targets into clinical practice and advance the prevention and management of neurological diseases.

## Conclusion

This review evaluated current evidence within an integrative framework, demonstrating that insulin resistance serves as a common pathophysiological substrate across diverse neurological disorders. Disruptions in central insulin signaling influence cognitive and behavioral functions through mechanisms such as dysregulated brain energy metabolism, increased neuroinflammation, impaired synaptic plasticity, and altered neuronal network balance. In conditions ranging from neurodegenerative diseases, including Alzheimer’s and Parkinson’s, to epilepsy and mood disorders, these mechanisms may accelerate disease onset and progression. Collectively, the evidence indicates that insulin resistance should be regarded not only as a metabolic abnormality but also as a central factor shaping the biological basis of neurological diseases.

Nutrition-based and metabolic interventions represent modifiable strategies that target this shared pathophysiological substrate. Dietary patterns such as the Mediterranean diet, low–glycemic load approaches, energy restriction, and ketogenic strategies have the potential to improve insulin sensitivity, reduce inflammation, and support brain energy utilization. The effectiveness of these interventions varies across individual metabolic phenotypes, underscoring the need for personalized approaches. Future longitudinal and rigorously designed clinical studies that clarify the effects of nutritional and lifestyle interventions on neurological outcomes are essential for defining the role of insulin resistance as a therapeutic target in the prevention and management of neurological diseases. In this context, nutrition-based interventions may be considered complementary yet transformative tools for addressing the metabolic dimension of neurological disorders.

## Data Availability

No datasets were generated or analysed during the current study.
